# *Ex vivo* generation of myeloid-derived suppressor cells that model the tumor immunosuppressive environment in colorectal cancer

**DOI:** 10.18632/oncotarget.3682

**Published:** 2015-03-29

**Authors:** Inès Dufait, Julia Katharina Schwarze, Therese Liechtenstein, Wim Leonard, Heng Jiang, David Escors, Mark De Ridder, Karine Breckpot

**Affiliations:** ^1^ UZ Brussel, Department of Radiotherapy, Vrije Universiteit Brussel, Brussels, Belgium; ^2^ Laboratory of Molecular and Cellular Therapy, Vrije Universiteit Brussel, Brussels, Belgium; ^3^ Navarrabiomed-Fundaçion Miguel Servet, Immunomodulation group, Pamplona, Spain; ^4^ Division of Infection and Immunity, University College London, London, UK

**Keywords:** MDSC, CRC, arginase-1, inducible nitric oxide synthase, GM-CSF

## Abstract

Myeloid-derived suppressor cells (MDSC) are a heterogeneous population of cells that accumulate in tumor-bearing subjects and which strongly inhibit anti-cancer immune responses. To study the biology of MDSC in colorectal cancer (CRC), we cultured bone marrow cells in conditioned medium from CT26 cells, which are genetically modified to secrete high levels of granulocyte-macrophage colony-stimulating factor. This resulted in the generation of high numbers of CD11b^+^ Ly6G^+^ granulocytic and CD11b^+^ Ly6C^+^ monocytic MDSC, which closely resemble those found within the tumor but not the spleen of CT26 tumor-bearing mice. Such MDSC potently inhibited T-cell responses *in vitro*, a process that could be reversed upon blocking of arginase-1 or inducible nitric oxide synthase (iNOS). We confirmed that inhibition of arginase-1 or iNOS *in vivo* resulted in the stimulation of cytotoxic T-cell responses. A delay in tumor growth was observed upon functional repression of both enzymes. These data confirm the role of MDSC as inhibitors of T-cell-mediated immune responses in CRC. Moreover, MDSC differentiated *in vitro* from bone marrow cells using conditioned medium of GM-CSF-secreting CT26 cells, represent a valuable platform to study/identify drugs that counteract MDSC activities.

## INTRODUCTION

Colorectal cancer (CRC) is characterized by the infiltration with various immune cell types [1]. The infiltration of CRC with CD8^+^ cytotoxic T lymphocytes (CTLs) has been correlated to a favorable prognosis [2, 3]. However, these CTLs are largely dysfunctional, as a result of their interaction with myeloid-derived suppressor cells (MDSC). Therefore, it was suggested that the absence of MDSC infiltration might serve as a better prognostic biomarker [4, 5]. Moreover, it was suggested that pharmacologic blockade of MDSC represents an attractive strategy to treat CRC.

Experimental CRC models such as those based on murine CT26 cells are often used to evaluate the growing list of anti-MDSC agents. This model is a valuable substitute for human CRC as, similarly to CRC in human patients, it is infiltrated with CTLs that are rendered inactive due to immunosuppression exerted by MDSC [6].

In mice, MDSC represent a heterogeneous population comprised of immature myeloid cells. These are characterized by the expression of CD11b and Gr-1, and lack markers specific for monocytes, macrophages and dendritic cells. MDSC can be subdivided in two subsets, namely monocytic and granulocytic MDSC on the basis of Ly6C-Ly6G expression profiles. While monocytic MDSC express low (or absent) Ly6G levels, granulocytic MDSC express high levels of Ly6G [7]. Various tumor-derived factors have been described to induce MDSC, these include but are not limited to granulocyte macrophage-colony stimulating factor (GM-CSF), macrophage-colony stimulating factor (M-CSF), prostaglandin E2 (PGE2), vascular endothelial growth factor (VEGF), stem cell factor (SCF), interleukin-6 (IL-6), IL-10 and IL-1β [7, 8]. Importantly, MDSC use a plethora of mechanisms to suppress antitumor immunity. One of these is the depletion of L-arginine mediated by arginase-1 (arg-1) and inducible nitric oxide synthase (iNOS), expressed in MDSC. L-arginine depletion was shown to limit T-cell proliferation and T-cell receptor signaling, and it is still considered the major mechanism through which MDSC mediate T-cell dysfunction [9, 10].

The ample evidence on the role of MDSC in cancers such as CRC has instigated research into the use of existing drugs as well as the development of novel drugs to deplete MDSC, block or revert their immunosuppressive activity. For example, chemotherapy drugs which have been shown to deplete MDSC include 5-fluororacil (5-FU) [11], gemcitabine [12, 13] and docetaxel [14]. In these drug discovery studies, MDSC derived from the spleen of tumor-bearing animals are most commonly used solely because they can be obtained in large numbers. However, splenic MDSC are phenotypically and functionally different from MDSC derived from within the tumor [15, 16]. Consequently, to ensure reliability and potency of novel MDSC-targeting drugs, they should be evaluated on tumor-derived rather than splenic MDSC. However, studying tumor-derived MDSC poses the technical challenge of obtaining sufficient number of cells at high purity from a limited number of tumor-bearing animals [17]. To circumvent this conundrum, researchers have evaluated various *in vitro* culture systems to obtain MDSC that closely resemble those found within the tumor. First of all, immortalized MDSC cell lines such as MSC-1 and MSC-2, were constructed using retroviral transduction but lack the distinct marker of MDSC, namely Gr-1 [18]. However, other *ex vivo* procedures starting from bone marrow cells were characterized by a low differentiation efficiency (up to 40%), resulting in only a limited amount of MDSC-like cells [19-27]. We recently developed an *ex vivo* system to efficiently differentiate bone marrow cells into MDSC [27, 28]. Herein conditioned medium from tumor cells that were transduced with lentiviral vectors encoding GM-CSF is used to differentiate bone marrow cells. A proof-of-concept on the value of this strategy to obtain large amounts of MDSC that resemble those found within B16 melanomas was delivered [28].

In the current study, we demonstrate that the *ex vivo* culture procedure is readily applicable to CRC and could be used as a predictive model as such facilitating the search for novel anti-MDSC drugs. Here we thoroughly characterize these *ex vivo* differentiated CRC-specific MDSC, demonstrate that their functions could be counteracted by arg-1 and iNOS inhibitors and that these treatments possess therapeutic activities *in vivo*.

## RESULTS

### High levels of GM-CSF are required to efficiently differentiate bone marrow cells to MDSC

**Figure 1:**
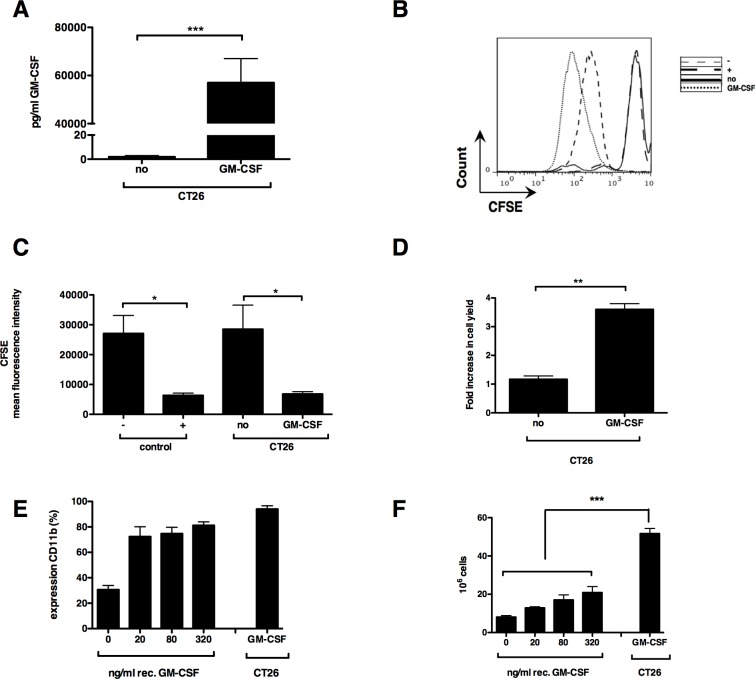
*Ex vivo* myelopoiesis can differentiate bone marrow cells into myeloid cells in the presence of GM-CSF (**A**) Graph representing murine GM-CSF content as measured by ELISA present in the CM of wildtype (no) and transduced (GM-CSF) CT26 tumor cells. (**B**) Representative histogram showing proliferation, as measured by dilution of CFSE, of the GM-CSF dependent FDCP-1 cells incubated for 72 hours in DMEM with (+) or without (−) recombinant GM-CSF (20 ng/ml) or incubated in CM of non-modified (no) and transduced CT26 tumor cells (GM-CSF). (**C**) Summarizing graph showing the mean fluorescence intensity (MFI) of CFSE positive FDCP-1 cells, a lower MFI representing strong proliferation of the FDCP-1 cells. (**D**) Fold increase in bone marrow cells incubated for 6 days in CM. (**E**) Expression of CD11b by bone marrow cells after a 6-day incubation period in CM. (**F**) Cell yield after 6 days incubation of 10 × 10^6^ bone marrow cells in CM. Mean of at least 3 experiments +/− SEM is shown in all graphs. Number of asterisks in the figures indicates the level of statistical significance as follows: *, *p* < 0.05; **, *p* < 0.01; ***, *p* < 0.001.

**Figure 2:**
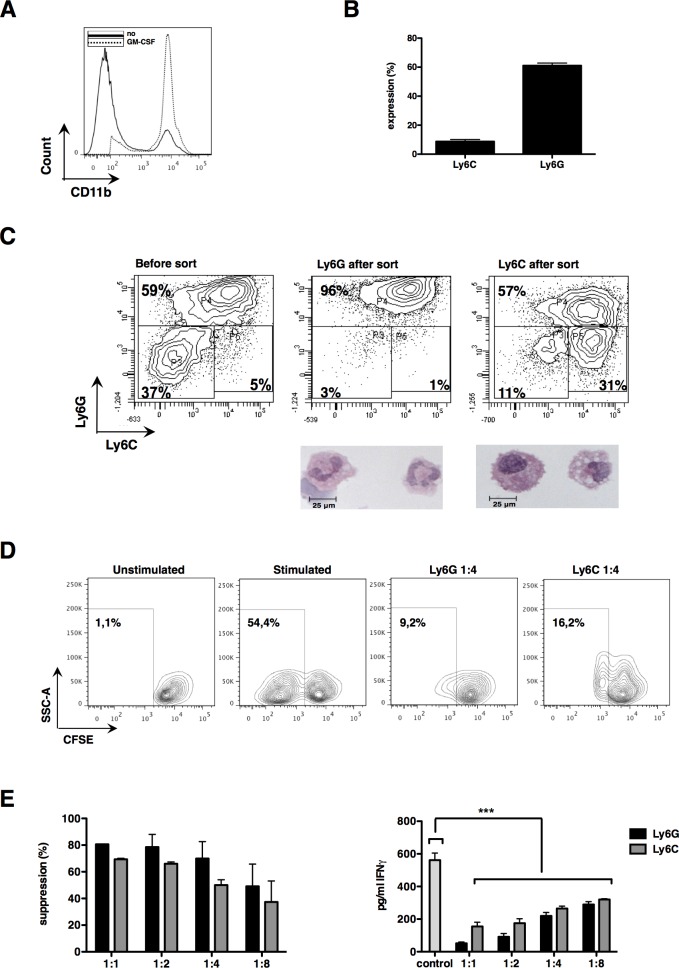
Differentiated bone marrow cells possess strong suppressive capacities and can be subdivided into both MDSC subsets (**A**) Expression of CD11b by bone marrow cells after a 6-day incubation period in CM as measured by flow cytometry. (**B**) Summarizing graph of ratio of MDSC subsets (**C**) Flow cytometry contour plots of *in vitro* MDSC before and after MACS sort. Underneath the contour plots of the sorted MDSC, representative pictures showing the morphology of these subsets are depicted. Pictures were taken with a light microscope at 64 times magnification. (**D**) Representative experiment showing suppression of CD8^+^ T cells by sorted *in vitro* MDSC (1:4 ratio MDSC to T cell). (**E**) The graph on the left represents the proliferation inhibition of CD3/CD28-activated CD8^+^ T cells (ratio MDSC to T cell as indicated in the graph). The graph on the right shows changes in IFN-γ secretion measured during the same experiment. Control represents T cells incubated without MDSC. Mean of at least 3 experiments +/− SEM is shown in all graphs. Number of asterisks in the figures indicates the level of statistical significance as follows: ***, *p* < 0.001.

CRC expands MDSC *in vivo*, which seem to contribute to tumor staging and poor prognosis [4, 29-31]. Usually, a large tumor burden is required to divert physiological myeloid differentiation towards MDSC expansion, possibly due to local and systemic GM-CSF accumulation. As we aimed to develop an *in vitro* culture system to differentiate bone marrow cells to MDSC resembling those found within CRC tumors, we first evaluated using ELISA whether the CRC cell line CT26 produced high levels of GM-CSF. CT26 tumor cells produced barely any GM-CSF (Fig. 1A). Therefore, we decided in analogy to our previous study on *in vitro*-generated melanoma MDSC [28], to transduce CT26 tumor cells with lentiviral vectors encoding GM-CSF. This resulted in secretion of high levels of GM-CSF (Fig. 1A). To examine whether the secreted GM-CSF was biologically active, GM-CSF-dependent FDCP-1 cells were labeled with CFSE and consequently cultured in the presence or absence of recombinant murine GM-CSF, as well as in conditioned medium (CM) of CT26-GM-CSF and CM of non-modified CT26 tumor cells. In this assay the proliferation of FDCP-1 cells incubated in recombinant GM-CSF was comparable to that of FDCP-1 cells incubated with CM of CT26-GM-CSF (Fig. 2B-2C). This CM was subsequently used to culture bone marrow cells, demonstrating that after 6 days of culture, cell yields were consistently higher in the high GM-CSF condition (Fig. 1D). Moreover, the majority of these cells expressed CD11b. This was not the case in cultures with CM of non-modified CT26 tumor cells (Fig. 1E). To identify the concentration of GM-CSF necessary to generate CD11b^+^ cells, we used CM of non-modified CT26 tumor cells supplemented with different concentrations of recombinant GM-CSF to culture bone marrow cells. High percentages of CD11b^+^ cells were generated in the presence of recombinant GM-CSF, without significant differences when using relative high (320 ng/ml) or low GM-CSF concentrations (20 ng/ml) (Fig. 1E). However, a significant difference was observed in the yield of CD11b^+^ cells between the conditions where recombinant GM-CSF or CM of transduced CT26 tumor cells was used (Fig. 1F). Since the yield and purity of CD11b^+^ cells was highest after differentiation in CM from CT26-GM-CSF cells (Fig. 2A), we continued with these culture conditions. MDSC are known to be a very heterogeneous population of cells but can be generally divided into a monocytic (Ly6C^+^) and a granulocytic (Ly6G^+^) subset. We examined the appearance of these subsets in the generated CD11b^+^ population (Fig. 2B). The ratio of the different subsets in the *in vitro* system coincides with the *in vivo* situation. Next, we examined their suppressive capacity as it is widely accepted that functionality and more specifically suppression of T-cell responses, is the single most important marker to identify MDSC. We showed that sorted CD11b^+^ Ly6C^+^ as well as CD11b^+^ Ly6G^+^ cells (Fig. 2C) had a high T-cell suppressive capacity (Fig. 2D-2E). Consequently, the CD11b^+^ cells obtained through the culture of bone marrow cells in CM of CT26-GM-CSF tumor cells could be considered as MDSC.

### *In vitro*-generated MDSC closely resemble MDSC found within both wild-type and GM-CSF-producing CT26 tumors

**Figure 3:**
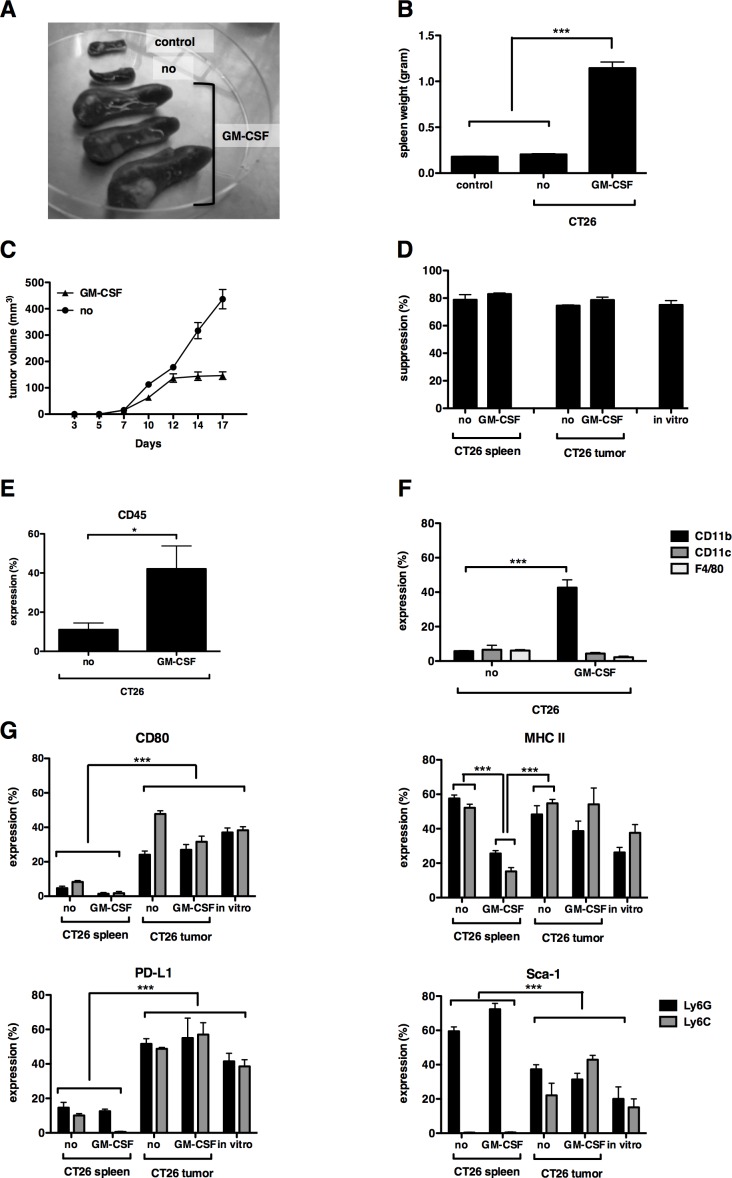
*In vitro*-generated MDSC closely resemble tumor-MDSC but not splenic-MDSC (**A**) Photograph of spleen of naive mice (control), CT26- (no) and CT26-GM-CSF (GM-CSF)-bearing mice at a tumor size of approximately 5 by 6 mm. (**B**) Summarizing graph showing spleen weight. (**C**) Tumor growth curve of CT26 versus CT26-GM-CSF-bearing mice. Day 1 represents the day of tumor injection. (**D**) Graph showing the proliferation inhibition of CD3/CD28-activated CD8^+^ T cells with bulk MDSC (1:1 MDSC to T cell ratio). (**E**) Summarizing graph showing CD45 infiltration in the tumor of CT26- versus CT26-GM-CSF-bearing mice. (**F**) Summarizing graph showing CD11b, CD11c and F4/80 content in the tumor of CT26- versus CT26-GM-CSF-bearing mice. (**G**) Summarizing graphs of different surface markers (CD80, MHC II, PD-L1 and Sca-1) present on MDSC, derived from spleen, tumor or *in vitro*-generated. Expression showed in the granulocytic (Ly6G) and monocytic (Ly6C) subset separately. Mean of at least 3 experiments +/− SEM is shown in all graphs. Number of asterisks in the figures indicates the level of statistical significance as follows: *, *p* < 0.05; ***, *p* < 0.001.

To examine whether the *in vitro*-generated MDSC resembled those found within CT26 tumors, we then compared their function and phenotype with MDSC isolated from the spleen and tumor of CT26-bearing mice. We also isolated MDSC from mice bearing CT26-GM-CSF tumor cells to examine the effect of GM-CSF overexpression *in vivo*. We observed that mice bearing CT26-GM-CSF tumors showed splenomegaly (Fig. 3A-3B) and moreover, that CT26-GM-CSF tumors hardly progressed after day 12, although the growth of unmodified CT26 or CT26-GM-CSF tumor cells initially followed a similar pattern (Fig. 3C). This decline in tumor growth was not correlated with the presence of tumor-specific T-cell responses as evaluated by ELISPOT (data not shown). In addition, evaluation of the T-cell suppressive activity of bulk (granulocytic and monocytic) MDSC showed that *in vitro*-generated MDSC, as well as MDSC derived from the spleen or tumor were highly capable of suppressing T-cell proliferation in a 1:1 MDSC to T cell ratio. No differences in functionality were observed between MDSC derived from CT26- and CT26-GM-CSF bearing mice (Fig. 3D). The CT26-GM-CSF tumors showed high infiltration of CD45^+^ cells, which correlated with a significant increase in CD11b^+^ but not CD11c^+^ or F4/80^+^ cells (Fig. 3E-3F).

Similar to previous reports [15, 16], we observed that MDSC found within the spleen (irrespective of the level of GM-CSF expression by CT26 cells) showed significant differences to MDSC found within the tumor. More specifically, a lower expression of CD80 and PD-L1 of splenic-MDSC was observed when compared to tumor-infiltrating MDSC (Fig. 3G). This data again confirmed the observation that MDSC accumulating in the spleen are distinct and different from tumor MDSC. Moreover, MDSC found within the spleen of mice bearing CT26-GM-CSF cells showed lower expression of MHC II when compared to MDSC obtained from tumors as well as the spleen of mice bearing non-modified CT26 cells. In contrast, lower expression of Sca-1 was observed in the granulocytic MDSC subset obtained from the tumor in comparison to the spleen, while opposite results were obtained for the monocytic MDSC subset. Importantly, the expression of MHC II, PD-L1 CD80 and Sca-1 was not significantly different on MDSC isolated from the tumor of non-modified CT26 cells when compared to CT26-GM-CSF tumors (Fig. 3G). The phenotype of *in vitro*-generated MDSC showed that they were closely related to the MDSC found within tumors but not spleen, as they expressed high levels of MHC II, PD-L1 and CD80 and low levels of Sca-1 (Fig. 3G). These results indicate that although high GM-CSF secretion impacts on the percentage and to a lesser extent phenotype of *in vivo* differentiated MDSC, the MDSC-T-cell suppressive activity is the same as found in mice bearing non-modified CT26 tumor cells. Moreover, these results show that our *in vitro*-generated MDSC phenotypically and functionally resemble the MDSC found within the tumor.

### The *in vitro*-generated MDSC are a reliable model to predict the outcome of MDSC-modulating drugs

**Figure 4:**
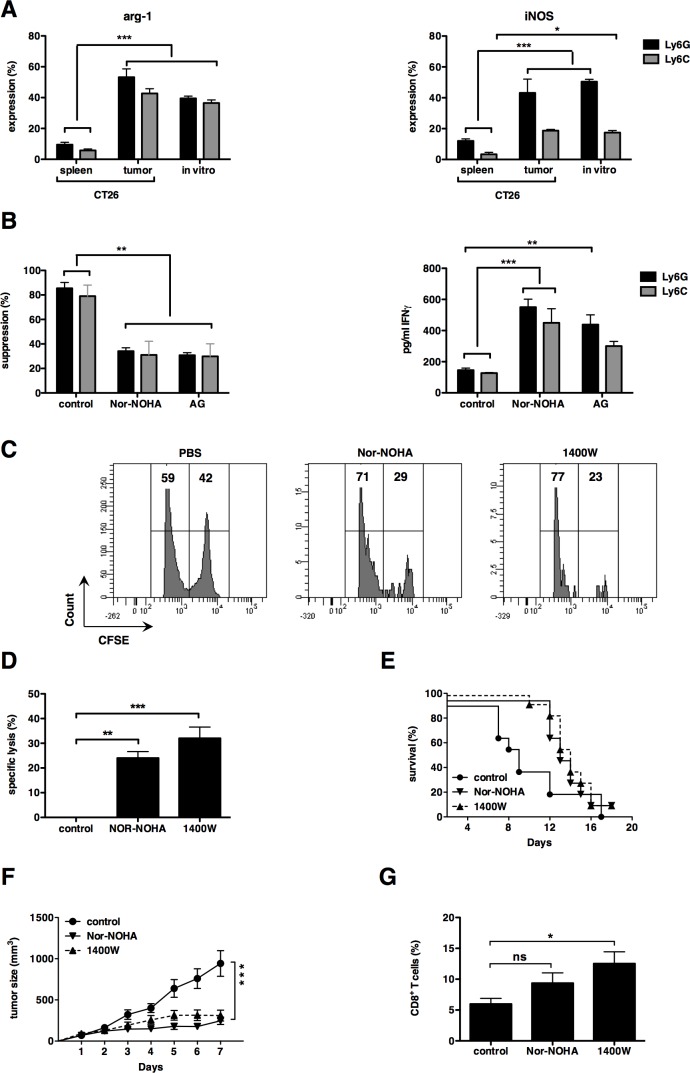
iNOS and arginase-1 can be used as targets to enhance cytotoxic T-cell responses (**A**) Summarizing graph representing arg-1 expression by MDSC at the left and iNOS expression by MDSC at the right. (**B**) The graph on the left shows the proliferation inhibition of CD3/CD28-activated CD8^+^ T cells with sorted *in vitro* MDSC (1:1 MDSC to T cell ratio). Control represents Cultures incubated without inhibitors. Cultures were supplemented with Nor-NOHA (300 μM) or AG (1 mM). The graph on the right shows changes in IFN-γ secretion measured during the same experiment. Mean of at least 3 experiments +/− SEM is shown in all graphs. (**C**) FACS graph showing the cytotoxic T-cell response against gp70 peptides of mice treated with Nor-NOHA, 1400W or PBS as a negative control. Number of mice per group = 3. (**D**) Summarizing graph of panel C. (**E**) Overall survival of treated mice. Mice were sacrificed when tumor diameter reached 15 mm. (**F**) Tumor growth curve of treated mice. Day 1 represents the first day of treatment, when tumor diameter reached approximately 6 mm. (**G**) Graph summarizing the percentage of CD8^+^ T cells present in the tumor of treated mice. Values are normalized to CD45 and CD3. Experiments were performed twice and included 6 mice per group. Mean +/− SEM is shown in all graphs. Number of asterisks in the figures indicates the level of statistical significance as follows: *, *p* < 0.05; **, *p* < 0.01; ***, *p* < 0.001.

A key aim of this study was to prove that the *in vitro*-generated MDSC can be used as a platform to predict the outcome of anti-MDSC drugs. In the literature, it is described that MDSC-mediated L-arginine depletion by the expression of arg-1 and iNOS, plays a major role in their T-cell suppressive capacity. Therefore, we chose inhibitors of arg-1 and iNOS as ideal candidates to test the predictive value of our *in vitro* MDSC-platform. Interestingly, arg-1 expression was high in tumor and *in vitro-*generated MDSC (both monocytic and granulocytic subsets) but not in splenic MDSC (Fig. 4A).

Similar iNOS expression was observed in the *in vitro*-generated and tumor CD11b^+^Ly6G^+^ granulocytic MDSC (Fig. 4A). iNOS expression was lower in *in vitro*-generated and tumor CD11b^+^Ly6C^+^ monocytic MDSC compared to their granulocytic counterparts, but still expressed to a higher extent then in splenic MDSC (Fig. 4A). These results confirm that the *in vitro*-generated MDSC closely resemble those found within CT26 tumors. To evaluate the extent to which arg-1 and iNOS contribute to the suppressive capacity of the *in vitro* MDSC, we performed an *in vitro* T-cell suppression assay with sorted CD11b^+^ Ly6G^+^ and CD11b^+^ Ly6C^+^ MDSC in the presence or absence of Nor-NOHA, an arg-1 inhibitor, and AG, an iNOS inhibitor. We showed that both the T-cell proliferation and IFN-γ production by the T cells was enhanced in the presence of these inhibitors (Fig 4B). These results confirmed the previously published role of arg-1 and iNOS in the T-cell suppressive activity of MDSC [7, 32] and suggest that the *in vitro*-generated CRC-specific MDSC are similar to MDSC obtained from CRC tumors. To evaluate the predictive value of CRC-specific MDSC, this was further confirmed *in vivo*, in which CT26-bearing mice were treated with Nor-NOHA or the specific iNOS inhibitor, 1400W, as the only treatment. We showed that treatment with either one of both inhibitors resulted in a CT26-specific cytotoxic immune response (Fig. 4C-4D). These data suggested that inhibition of arg-1 or iNOS enabled CD8^+^ cytotoxic T cells to escape the MDSC-mediated immune suppression, although overall survival of treated groups was not significantly prolonged (Fig. 4E). Moreover, a delay in tumor growth was observed as long as both inhibitors were administered (Fig. 4F). Comparison of the CD8^+^ T-cell infiltration of tumors of non-treated mice or mice treated with Nor-NOHA or iNOS, showed that the number of CD8^+^ T cells was enhanced in both treatment conditions (Fig. 3G). These data show that inhibition of arg-1 and iNOS resulted in higher numbers of functional CD8^+^ cytotoxic T cells, as predicted in the *in vitro* T-cell suppression assay using *in vitro-*generated CRC-specific MDSC.

## DISCUSSION

GM-CSF is one of the most important factors produced by tumor cells leading to MDSC expansion. In literature, it is evident that GM-CSF plays an important role in the accumulation of MDSC [7, 20, 33]. That is why current *ex vivo* MDSC differentiation protocols primarily rely on culturing bone marrow hematopoietic progenitors with recombinant GM-CSF. But clearly, other still unknown factors contribute to MDSC differentiation and expansion, as efficiency rarely surpasses 40%, even with the addition of various other cytokines, such as IL-4, IL-13, PGE2, [21, 24, 26].

In this study, we demonstrated the feasibility of generating *in vitro* MDSC in a CRC model using the system described by Liechtenstein *et al.* in a melanoma model [28]. They reasoned that endogenous GM-CSF could have better differentiation efficiency as myelopoiesis within a tumor microenvironment was simulated. Obviously, this system does not mimic the complexity of the *in vivo* situation, but it may be a good practical approximation. Indeed, differentiation efficiency up to 90% was achieved in our CRC model, while maintaining high proliferation capacity. Another advantages of this protocol compared to previously described methods are the high MDSC yields, which can not be obtained by merely supplementing CM of CT26 cells with recombinant GM-CSF. Importantly, the high yield of pure CD11b^+^ cells obtained in this culture system circumvents the need to grow tumors and sacrifice a large number of mice to obtain sufficient tumor MDSC. Other advantages of the method presented in this manuscript, is its reproducibility and the ease at which this technique can be performed.

The one true accepted marker of MDSC is their suppressive capacity, as these cells otherwise display a great heterogeneity [34-37]. Our *in vitro*-generated CRC-specific MDSC are very potent immunosuppressive cells, demonstrated by their ability to suppress T cells, even at a 1:8 MDSC to T cell ratio. In addition, our data confirmed that the *in vitro*-generated CRC-specific MDSC are more similar to tumor MDSC than splenic MDSC. Therefore, this model would be more relevant in drug discovery studies than splenic MDSC. Spleen-derived MDSC are still widely used in MDSC research [38-43], despite the proof that these cells are very different indeed, both phenotypically as functionally [15].

The primary aim of the *ex vivo* MDSC generation protocol is to use these cells as a predictive tool for high-throughput screening in the search for new anti-MDSC drugs. However, its use is not restricted and they could be applied in a very broad manner. For example, Van der Jeught *et al.* used this *ex vivo* differentiation protocol to examine the modulation of the tumor microenvironment by using mRNA encoding soluble proteins [44].

In this study, we have shown that GM-CSF overexpression *in vivo* does initially not lead to changes in tumor volume, but does, amongst others, cause splenomegaly. Similar abnormalities were previously described in GM-CSF transgenic mice [45-47]. The increased number of myeloid cells in tumor and spleen is characterized by a CD11b^+^ MDSC population and can be seen as an immune-inhibitory infiltrate [48]. These findings are consistent with the study performed by Bronte *et al.* who showed that a population of suppressive CD11b^+^/Gr-1^+^ cells increased when tumor cells were modified to produce GM-CSF [33]. However, there is no clear consensus about the effects of chronic GM-CSF expression on tumor growth, as studies have shown either an anti-proliferative effect [49, 50], a tumor-promoting effect [51, 52] or no significant effect on tumor growth rate [53, 54]. We showed no significant effects during the first 12 days of tumor growth. Long-lasting follow-up of tumor growth was impossible in our study, as the mice had to be sacrificed due to their splenomegaly, the latest at day 17. Nonetheless, we observed that the size of CT26-GM-CSF tumors remained stable from day 12 onwards, whereas CT26 tumors continued to grow. To our surprise the lack of continued growth of CT26-GM-CSF tumors was not correlated to a tumor-specific T-cell response, suggesting that other mechanisms are responsible for the tumor control. Although, chronic GM-CSF expression was shown in some studies to lead to malignant progression of the tumor due to enhanced angiogenesis, invasiveness and migration [55-57], other studies showed that GM-CSF production can also lead to improved survival in CRC [58]. GM-CSF has been studied for a while as a vaccine adjuvant in cancer immunotherapy due to its immunostimulatory properties [47, 59]. However, the results in clinical trials were disappointing in terms of immune responses and clinical outcome [60, 61]. No link between GM-CSF-induced MDSC expansion and failure of GM-CSF in clinical trials has been demonstrated, but caution in this field is required as GM-CSF is much more then solely an immunostimulating cytokine.

Well-studied amino acid-consuming enzymes in MDSC biology are arg-1 and iNOS, both present on the two MDSC subtypes and important in conferring immunosuppressive capacities to these cells [7, 36]. As these molecules are important in the basis MDSC biology, we used them to further examine our *in vitro*-generated CRC-specific MDSC. Consistent with *in vivo* MDSC [10, 21, 62], inhibiting arg-1 and iNOS directly affects their T-cell immunosuppressive activities. Furthermore, we inhibited arg-1 or iNOS intratumorally to examine whether we could observe similar effects in comparison to the *in vitro* system. To this end, we used the inhibitors Nor-NOHA and 1400W, respectively. AG was replaced with 1400W to serve as the iNOS inhibitor during the *in vivo* study, since AG is a general NOS inhibitor, namely an inhibitor of iNOS but also endothelial NOS and neuronal NOS. The latter are not present in the *in vitro* culture, while 1400W specifically inhibits iNOS and is better suited for an *in vivo* setting. During treatment, a significant delay in tumor growth was observed and cytotoxic T-cell responses increased. In contrast, another study using a different arg-1 inhibitor, namely N(G)-nitro-L -arginine methyl ester (L-NAME), reported no increase in endogenous antitumor immunity [63]. Despite a significant increase in cytotoxic T cells, differences observed in CD8^+^ T cells were not as pronounced as anticipated. The lack hereof can probably be attributed to the experimental design. Treatment was ceased at day 10 and subsequent tumor growth might have allowed the ”reconstitution” of the tumor environment. This experimental setup was chosen, as initially we were more interested in tumor development and overall survival. Another possibility is that inhibition of MDSC immunosuppressive activities may not alter the infiltration of immune cells (which would depend on cell trafficking), but rather their antitumor properties. We strongly believe that the immune signature of the tumor during treatment differs, as characterized by the increase in cytotoxic T lymphocytes during treatment. No inhibition in tumor growth was observed when similar experiments, in which arg-1 was inhibited, were performed in mice lacking functional T and B cells [64]. This suggests that the growth delay of the tumor caused by the inhibition of arg-1 is at least partially dependent on the immune system. We are also aware that only one mechanism, either arg-1 or iNOS, is studied in this setting. Still, we can modulate the tumor environment leading to enhanced antitumor T cell responses. We believe that these T-cell responses could be further potentiated through combination therapy, ideally a mix of inhibitory (for example anti-MDSC drugs) and immunostimulatory molecules (for example vaccination).

The predictive value of *in vitro* MDSC still has to be examined further, but preliminary results already give a good indication about future possibilities of this *ex vivo* differentiation system.

## MATERIALS AND METHODS

### Mice and cell lines

Female, 6 to 8 weeks old Balb/c mice (Charles River Laboratories, L'Arbresle Cedex, France) were treated according to the European guidelines for animal experimentation. Experiments were reviewed by the Ethical Committee for use of laboratory animals of the Vrije Universiteit Brussel (Jette, Belgium). The mouse colon cancer cell line CT26 and mouse lymphoblast cell line FDCP-1 were obtained from the American Type Culture Collection (ATCC, Molsheim Cedex, France) and cultured according to the recommendations of ATCC.

### Production and characterization of lentiviral vectors encoding GM-CSF

The packaging plasmid pCMVΔR8.9 and VSV.G encoding plasmid pMD.G were a gift from Dr. D. Trono (University of Geneva). The plasmid encoding GM-CSF and the puromycin resistance gene was previously described [28]. The production and characterization of lentiviral vectors was performed as described before [65].

### Transduction of CT26 cells with lentiviral vectors encoding GM-CSF

Tumor cells, namely CT26, which over express GM-CSF, were generated by transduction with lentiviral vectors encoding for both mouse GM-CSF and the puromycin resistance gene. To that end 2 × 10^5^ CT26 cells were plated in 2 ml culture medium in a 6-well. One day later, the culture medium was replaced with 2 ml of the lentiviral transduction cocktail containing 15 infectious lentiviral particles per cell and 10 μg/ml protamine sulphate (Leo Pharma, Lier, Belgium). Three days later, transduced cells were selected using 3 μg/ml puromycin (Sigma-Aldrich, Diegem, Belgium). To collect conditioned medium (CM), cells were plated at 10 × 10^6^ cells in 25 ml culture medium in a T175 cm^2^ and kept in culture in the absence of puromycin for 3 days. To verify the production of GM-CSF, CM was used to culture FDCP-1 cells. To quantify the amount of GM-CSF, an ELISA (eBioScience, Vienna, Austria) was performed according to manufacturer's instructions.

### *In vitro*-differentiation of myeloid-derived suppressor cells

Bone marrow cells were extracted from the femur and tibia of Balb/c mice, after which 10 × 10^6^ bone marrow cells were cultured in 75% CM and 25% Iscove's Modified Dulbecco's medium (IMDM, Sigma-Aldrich) supplemented with 10% fetal clone I (FCI, GE Health Care Life Sciences, Hyclone Laboratories, Utah, USA), 100 U/ml penicillin, 100 μg/ml streptomycin (Sigma-Aldrich) and 2 mM L-glutamine (Sigma-Aldrich) for 6 days. Cell viability and cell numbers were evaluated by trypan blue staining (Sigma-Aldrich). To evaluate the morphology of the cells, 5 × 10^5^ sorted MDSC were fixed on glass slides using the cytospin technique and were centrifuged at a speed of 1000 rpm for 5 minutes. Cytospin slides, filter cards, sample chambers, and metal clips were all obtained from Thermo scientific (Massachusetts, USA). Cytospins were air dried for 2 hours and afterwards stained with hematoxylin and eosin.

### Isolation of *in vivo* differentiated myeloid-derived suppressor cells

In order to grow tumors, Balb/c mice received a subcutaneous injection of 1 × 10^5^ CT26 tumor cells. When the tumor diameter exceeded 15 mm, mice were sacrificed and single cell suspensions from the tumor and spleen were obtained as previously described [66]. To enrich Ly6G^+^ or Ly6C^+^ MDSC, we sorted the MDSC using the Myeloid-Derived Suppressor Cell Isolation Kit according to the manufacturer's instructions (Miltenyi Biotec, Bergisch-Gladbach, Germany).

### Cell staining and flow cytometry

Staining of cell surface markers was performed as described [67]. The following antibodies were used: anti-CD11b-eF450, anti-MHC II-PE, anti-F4/80-APC-H7 (eBioScience), anti-CD11b-FITC, anti-Ly6G-AF647, anti-Ly6C-Pe-Cy7, anti-CD80-BV421, anti-PD-L1-PE, anti-CD3-PercP-Cy5.5, anti-CD11c-AF647 (Biolegend, London, United Kingdom), anti-Ly6G-PE-CF594, anti-CD8-FITC (Becton Dickinson, Erembodegem, Belgium) and anti-CD45-VioBlue (130-102-775) (Mitenyi Biotec). For intracellular staining, cells were treated with inside FIX and incubated for 20 minutes at room temperature. Cells were incubated with PERM (Miltenyi Biotec) and the anti-arginase-1-PE (R&D systems, Abingdon, United Kingdom) or anti-iNOS-PercP-Cy5.5 (Santa Cruz Biotechnology, Heidelberg, Germany) antibody for 20 minutes at room temperature. Subsequently, cells were washed. Cells stained with isotype matched control antibodies served as a control. Cells were acquired using the LSR Fortessa (Becton Dickinson) and analysis was performed using FlowJo 7.6 (Treestar Inc Oregon, United States of America).

### *In vitro* T-cell suppression assay

To evaluate the suppressive activity of MDSC, we performed an *in vitro* T-cell suppression assay. To that end, CD8^+^ T lymphocytes were isolated from the spleen of Balb/c mice using the CD8α^+^ T cell Isolation Kit II (Miltenyi Biotec). These CD8α^+^ T lymphocytes were labeled with carboxyfluorescein diacetate succinimidyl ester (CFSE, Life Technologies, Gent, Belgium). First, cells were washed and suspended in 5 ml phosphate buffered saline (PBS, Sigma-Aldrich) containing 0.1% bovine serum albumin (BSA, Life Technologies). Five ml of 0.5 μM CFSE were added to the cell suspension. The single cell suspension was incubated at 37°C, 5% CO_2_ for 10 minutes, washed in serum free Optimem (Invitrogen, Life Technologies), centrifuged for 7 minutes at 1500 rpm and suspended in 5 ml Optimem. Cells were plated at 1 × 10^5^ cells in 100 μl in a 96-well. Subsequently, the cells were either left unstimulated or were stimulated with a 1/800 dilution of anti-CD3/anti-CD28 coated beads (Invitrogen). Enriched Ly6G^+^ or Ly6C^+^ MDSC were obtained using the Myeloid-Derived Suppressor Cell Isolation Kit (Miltenyi Biotec). Sorted MDSC were added to the stimulated T cells at the indicated MDSC to T cell ratios. When indicated, specific inhibitors for arg-1 (Nω-hydroxy-nor-Arginine (Nor-NOHA), 300 μM) (Enzo Life Sciences, Antwerpen, Belgium) or iNOS (aminoguanidine (AG), 1 mM) (Sigma-Aldrich) were added. Dilution of CFSE was evaluated 3 days later by flow cytometry as a measure of T-cell proliferation. To that end, T cells were additionally stained with anti-CD3-PercP-Cy5.5 (Biolegend). Data were collected using the FACSCanto Flow Cytometer (Becton Dickinson) and were analyzed with FlowJo 7.6 (Treestar Inc.). During the analysis, cells were gated according to their forward and side scatter distribution and to CD3 expression. Alternatively, supernatants were collected and screened for IFN-γ content using a standard ELISA (Thermo scientific) according to manufacturer's instructions.

### Therapy

Balb/c mice received a subcutaneous injection of 1 × 10^5^ CT26 tumor cells. When the tumors reached a diameter of 6 mm, mice were treated for 10 consecutive days with an intratumoral injection of 50 μl Nor-NOHA (80 mg/kg) (Enzo Life Sciences), 1400W hydrochloride (20 mg/kg) (Sigma- Aldrich) or PBS. The tumor volume was measured on a daily basis using a caliper. Mice were sacrificed by neck dislocation when the tumor diameter exceeded 15 mm.

### *In vivo* cytotoxicity assay

An *in vivo* cytotoxicity assay was performed after 5 consecutive treatments with Nor-NOHA, 1400W or PBS (see above) to evaluate the stimulation of cytotoxic antitumor immune responses. The assay was performed as described by Van Lint *et al.* [68] using gp70 peptide (Thermo Electron GmbH, Ulm, Germany) pulsed cells as targets.

### ELISPOT

Lymph nodes (LN) and spleens were isolated and single cell suspensions were prepared as described before by Goyvaerts *et al.* [66]. Enzyme-linked immunospot (ELISPOT) plates (Millipore, Brussels, Belgium) were coated with 100 μl purified anti-IFN-γ antibodies and incubated overnight at 4°C. Wells were then blocked with 100 μl blocking buffer (RPMI supplemented with 10% FCI). A total of 1 × 10^5^ MACS sorted CD8^+^ splenocytes (Miltenyi Biotec.) or 2 × 10^5^ unsorted LN cells were plated per well (in duplicate). Cells were either left unstimulated and served as a negative control or cells were treated with gp70 peptide. Concanavalin A (Sigma- Aldrich) stimulated T cells served as a positive control. ELISPOT plates were incubated at 37°C, 5% CO for 24h. Next, the ELISPOT plates were developed according to the manufacturer's instructions (Diaclone, Besançon, France). Spots were counted using an ELISPOT counter (Autoimmun Diagnostika GmbH, Straβberg, Germany) and software (Autoimmun Diagnostika ELISPOT Reader 5.0).

### Statistical analyses

A t-test and a one-way ANOVA followed by a Bonferroni's multiple comparison test was performed. Sample sizes and number of times experiments were repeated are indicated in the figure legends. Number of asterisks in the figures indicates the level of statistical significance as follows: *, *p* < 0.05; **, *p* < 0.01; ***, *p* < 0.001. The results are shown in column graphs as the mean ± standard error of the mean (SEM). Survival of mice in the therapy experiment was visualized in a Kaplan-Meier plot. Differences in survival were analyzed by the log-rank test. All data were analyzed using GraphPad Prism 5.
